# On the role of Ce in CO_2_ adsorption and activation over lanthanum species†
†Electronic supplementary information (ESI) available. See DOI: 10.1039/c8sc00203g


**DOI:** 10.1039/c8sc00203g

**Published:** 2018-02-23

**Authors:** Xinyu Li, Zhi-Jian Zhao, Liang Zeng, Jiubing Zhao, Hao Tian, Sai Chen, Kang Li, Sier Sang, Jinlong Gong

**Affiliations:** a Key Laboratory for Green Chemical Technology of Ministry of Education , School of Chemical Engineering and Technology , Tianjin University , Tianjin 300072 , China . Email: jlgong@tju.edu.cn; b Collaborative Innovation Center of Chemical Science and Engineering (Tianjin) , Tianjin 300072 , China

## Abstract

La_2_O_3_ exhibits good performance for various catalytic applications, such as oxidative coupling of methane (OCM) and dry reforming of methane (DRM), during which coke formation may lead to the deactivation of catalysts. Typically, the reaction between CO_2_ adsorbed on La_2_O_3_ and coke is the rate-determining step of the coke elimination process. This paper describes the influence of Ce addition on the CO_2_ adsorption and activation over La_2_O_3_. Combined with *in situ* and *ex situ* characterization and density functional theory (DFT) calculation, we show that Ce addition promotes the formation of bidentate carbonate on La_2_O_3_*via* tuning CO_2_ adsorption energy. In addition, Ce addition adjusts the ratio of bidentate/monodentate carbonate, and affects the ratio of hexagonal/monoclinic La_2_O_2_CO_3_ on the binary oxides. DRM is used as a probe reaction to examine the coke elimination performance of Ce–La binary oxide. It is found that when the Ce/La ratio reaches the optimal value (0.15), Ce–La binary oxide has the highest CO_2_ adsorption energy and predominantly promotes the formation of bidentate carbonate, and hence possesses the highest basicity above 700 °C and finally exhibits the best coke elimination performance.

## Abstract 2

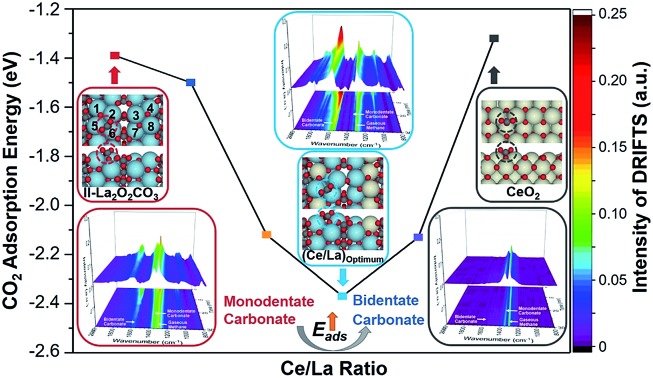
This paper describes the influence of Ce addition on the CO_2_ adsorption and activation over La_2_O_3_. Ce addition is verified to promote the formation of bidentate carbonate on La_2_O_3_ and affect the ratio of hexagonal/monoclinic La_2_O_2_CO_3_ on the Ce–La binary oxides.

## Introduction

La_2_O_3_, as a common rare earth oxide with strong basicity, is generally used as a support or promotor to facilitate CO_2_ adsorption and activation.^1^–^4^ Typically, CO_2_ adsorbs on the surface of La_2_O_3_ and subsequently converts La_2_O_3_ to La_2_O_2_CO_3_.^5^,^6^ The formed La_2_O_2_CO_3_ species actively reacts with coke thus enhancing catalyst stability.^5^–^8^ Additionally, La_2_O_2_CO_3_ plays a significant role in the oxidative coupling of methane (OCM), and CH_4_ can be activated over the surface of La_2_O_2_CO_3_.^9^,^10^ La_2_O_2_CO_3_ has three phases including tetragonal (I type), monoclinic (Ia type), and hexagonal (II type).^11^,^12^ As treatment temperature increases, phase transformation happens. Transformation from I-La_2_O_2_CO_3_ to Ia-La_2_O_2_CO_3_ takes place readily, namely Ia-La_2_O_2_CO_3_ is the monoclinic distortion of I-La_2_O_2_CO_3_.^12^,^13^ Conversion of Ia-La_2_O_2_CO_3_ to II-La_2_O_2_CO_3_ is a slow process below 600 °C.^12^ And II-La_2_O_2_CO_3_ decomposes to La_2_O_3_ at about 750 °C.^14^ It has been reported that the orientation of carbonate ions is closely related to the crystalline phase of La_2_O_2_CO_3_,^11^,^15^ both of which can affect the catalytic performance.^16^,^17^ It has been found that mixing ZnO with La_2_O_2_CO_3_ increases the basicity of the La_2_O_2_CO_3_ material.^18^ In addition, Metiu *et al.* reported that dopants can affect CH_4_ activation and dissociation on lanthanum oxide and hence improve the OCM performance.^19^,^20^

Generally, ceria possesses good redox properties and has various applications.^21^,^22^ It has been extensively used as an oxygen carrier^23^ and is a necessary component of catalysts used in reforming processes,^24^–^26^ water–gas shift reaction,^27^ CO oxidation,^28^ and soot combustion.^29^,^30^ In order to improve the oxygen storage capacity (OSC) and oxygen mobility (OM) of ceria, an appropriate dopant is typically mixed with ceria to enhance the OSC/OM of ceria.^31^–^33^ La^3+^, as an aliovalent dopant, has been extensively applied to enhance the OSC/OM of ceria,^34^–^36^ during which oxygen vacancies can be formed due to the charge compensation mechanism.^37^,^38^ It should be noted that the reported synergy of Ce–La binary oxide is based on the fact that La addition can largely promote the formation of oxygen vacancy on ceria,^36^–^38^ while this paper investigates the influence of Ce addition on the properties of lanthanum species.

Since the release or uptake of lattice oxygen is closely related to an oxygen/steam atmosphere, herein an oxygen/steam atmosphere is excluded to minimize the involvement of oxygen vacancy. Thus, CO_2_ as a soft oxidant is selected due to its weak oxidation capacity compared with O_2_ or H_2_O molecule. Given that CO_2_ adsorbs on the surface of La_2_O_3_ and reacts with La_2_O_3_ to form La_2_O_2_CO_3_,^5^,^6^ the effect of Ce addition on the properties of La_2_O_2_CO_3_ formed under the mild oxidative conditions has been investigated. For the OCM reaction, coke deposition is negligible in an oxidative atmosphere, but it takes place under oxygen lean conditions.^39^,^40^ When O_2_ is replaced by CO_2_ to rule out the influence of oxygen vacancy, dry reforming of methane (DRM) mainly occurs, during which CH_4_ reacts with CO_2_ to form syngas (CO and H_2_). For the DRM reaction, coke deposition and sintering of metal particles can lead to the deactivation of catalysts.^4^,^41^ Herein, the DRM reaction is used as a probe reaction to examine the coke elimination performance of Ce–La binary oxides.

This paper demonstrates the influence of Ce addition on the properties of lanthanum species, including the adsorption mode of CO_2_ (bidentate carbonate and monodentate carbonate) and the crystalline phase of lanthanum oxycarbonate (hexagonal La_2_O_2_CO_3_ and monoclinic La_2_O_2_CO_3_) formed after CO_2_ and CH_4_ adsorption. *In situ* diffuse reflectance infrared Fourier transform spectroscopy (DRIFTS) measurements are applied to investigate surface species on the Ce–La binary oxide during the process of CO_2_/CH_4_ adsorption. The physical–chemical properties of the catalysts prior to and after CO_2_/CH_4_ adsorption are investigated by X-ray diffraction (XRD), X-ray photoelectron spectroscopy (XPS), Raman spectra, N_2_-physisorption, transmission electron microscopy (TEM), and H_2_ temperature-programmed reduction (H_2_-TPR). Periodic density functional theory (DFT) calculations are carried out to estimate CO_2_ adsorption energy on Ce–La binary oxides. DRM is selected as the probe reaction to examine the performance of coke elimination, and CO_2_ temperature-programmed desorption (CO_2_-TPD) and thermogravimetric analysis (TGA) are applied to examine the basicity of Ce–La binary oxides and properties of the deposited coke during the DRM process.

## Results and discussion

### Textural properties of Ce–La binary oxide

**Fig. 1:**
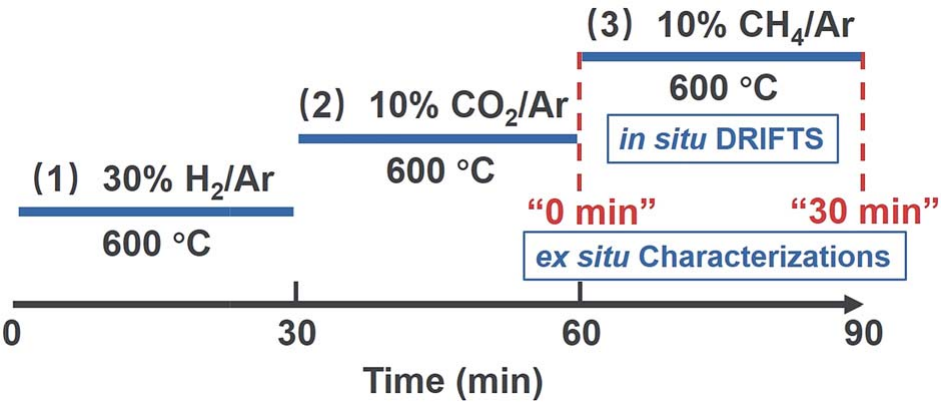
Overview of the experimental method for *in situ* and *ex situ* reaction conditions during measurement.

**Fig. 2:**
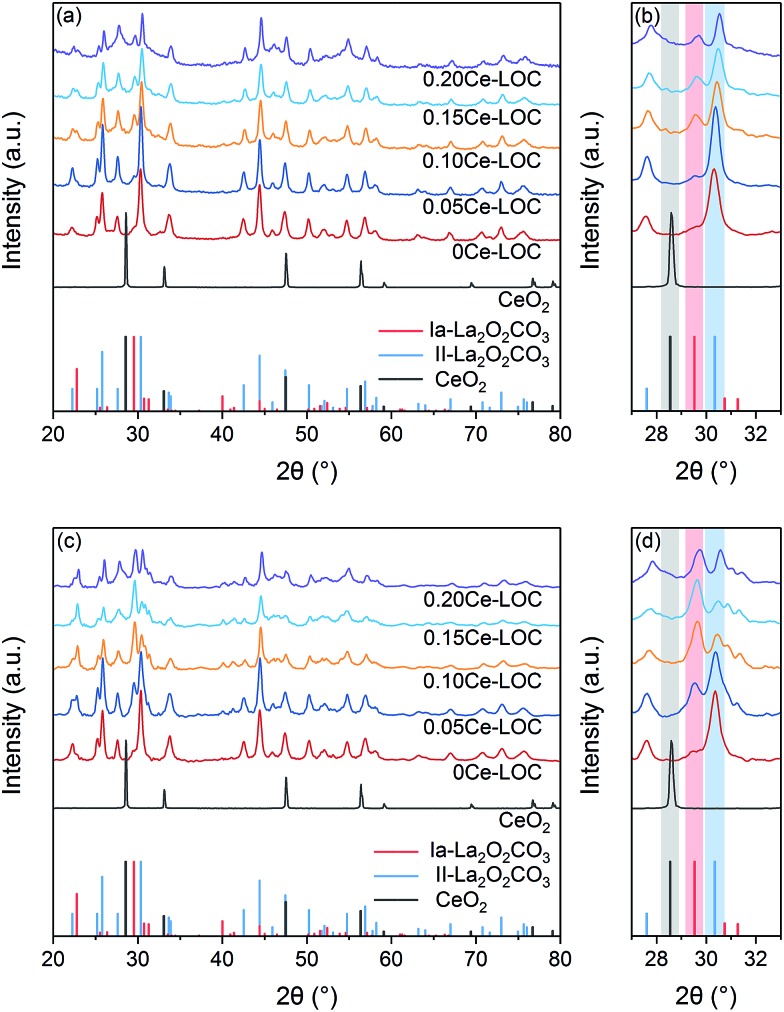
XRD patterns of the series of Ce–La binary oxide prior to and after CH_4_ adsorption for 30 min. (a) Full scale at “0 min”, (b) details with enlarged scale at “0 min”, (c) full scale at “30 min” and (d) details with enlarged scale at “30 min”. Color bars in (b) and (d) are auxiliary lines.

Table 1 sums up the textural properties of the samples with different Ce/La ratios at the moment of “0 min” (Fig. 1, see details in the Experimental section). XRD patterns of a series of Ce–La binary oxides at “0 min” are shown in Fig. 2a and b. Hexagonal lanthanum oxycarbonate (II-La_2_O_2_CO_3_, JCPDF#37-0804) acts as the dominant species for the series of Ce–La binary oxides. When the Ce/La ratio reaches 0.10 and higher, monoclinic lanthanum oxycarbonate (Ia-La_2_O_2_CO_3_, JCPDF#48-1113) can be detected but is minimal. Results show that the peaks of La_2_O_2_CO_3_ gradually migrate to a higher degree (Fig. 2b), indicating that lattice parameters decrease correspondingly (Table 1). Since the ionic radius of either Ce^3+^ (0.101 nm) or Ce^4+^ (0.097 nm) is smaller than that of La^3+^ (0.11 nm),^37^ the decrease of lattice parameters could be attributed to the fact that Ce-ions are doped into the lattice of La_2_O_2_CO_3_.^42^ Furthermore, the surface areas of the series of Ce–La binary oxides were obtained through the N_2_ sorption isotherm method. With the increase of Ce/La ratios, surface area gradually decreases, which is related to the decrease of lanthanum composition in the binary oxide and the small surface area of the cerium composition. The types of carbonate, formed upon CO_2_ adsorption, have a close relationship with the crystalline phases of La_2_O_2_CO_3_, which will be discussed in the following part.

**Table 1:** Textural properties of the series of Ce–La binary oxide

Sample	Molar ratio of Ce/La		Lattice parameter^*c*^ (Å)		Surface area^*d*^ (m^2^ g^–1^)	Average pore diameter^*d*^ (nm)	Pore volume^*d*^ (cm^3^ g^–1^)
	Bulk^*a*^	Surface^*b*^	*a*	*b*			
0Ce–LOC	0	0	4.078	15.950	47	28.4	0.22
0.05Ce–LOC	0.049	0.21	4.075	15.940	43	16.1	0.20
0.10Ce–LOC	0.102	0.18	4.074	15.933	33	14.4	0.14
0.15Ce–LOC	0.149	0.23	4.068	15.923	25	11.6	0.09
0.20Ce–LOC	0.203	0.22	4.065	15.854	14	10.0	0.08

^*a*^Determined by ICP-OES.

^*b*^Determined by XPS.

^*c*^Determined by XRD patterns prior to CH_4_ adsorption.

^*d*^Determined by N_2_-physisorption.

**Fig. 3:**
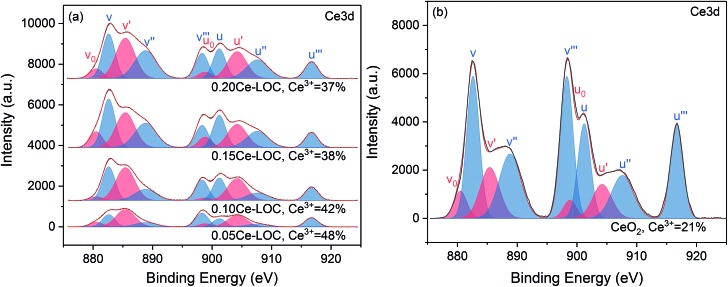
Fitting of core-level Ce 3d XPS profiles of (a) the series of Ce–La binary oxide and (b) CeO_2_.

The valence state of cerium (Fig. 3) and the surface elemental composition (Table 1) were also examined. On the basis of literature reports,^43^–^47^ the Ce 3d region consists of five doublets. The spin–orbit components with unprimed labels, v and u, are ascribed to the primary Ce 3d_5/2_ and Ce 3d_3/2_ states while other doublets represent satellite features arising from the Ce 3d_5/2_ and Ce 3d_3/2_ ionization.^44^,^46^,^48^ The doublets labeled *v*_0_/*u*_0_ and *v*′/*u*′ are characteristic of Ce^3+^, while the remaining doublets labeled *v*/*u*, *v*′′/*u*′′ and *v*′′′/*u*′′′ are characteristic of Ce^4+^.^44^,^46^ The Ce^3+^ surface concentration was calculated *via* the following equation:^44^,^48^

1

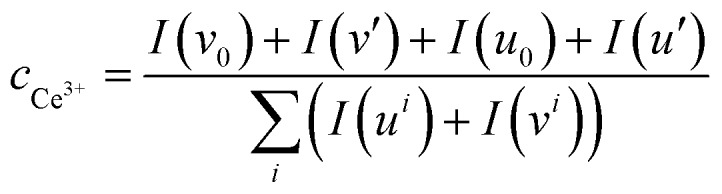

where *c*_*x*_ denotes the concentration of *x* and *I*(*y*) denotes the integral intensity of the specific peak. It should be noted that the Ce^3+^ content of CeO_2_ is 21% while other Ce–La binary oxides have much higher Ce^3+^ content than CeO_2_ (Fig. 3). It is assumed that the enhanced content of Ce^3+^ is doped into the lattice of La_2_O_2_CO_3_ to maintain the concentration of Ce^3+^, which is consistent with the reduced lattice parameter. As the Ce/La ratio increases from 0.05 to 0.20, the Ce^3+^ content gradually decreases (Fig. 3), indicating that the trend to be doped into the lattice of La_2_O_2_CO_3_ is close to saturated state and Ce^4+^ might exist in the form of CeO_2_ distributed on the surface of the binary oxide. Additionally, CeO_2_ diffraction peaks are absent even in the enlarged graph of XRD patterns (Fig. 2b), which suggests that CeO_2_ particles are uniformly dispersed on the surface of the Ce–La binary oxide. For the series of Ce–La binary oxides (Table 1), bulk Ce/La ratios obtained from ICP-OES were consistent with nominal Ce/La ratios, while surface Ce/La ratios obtained from XPS are higher than bulk Ce/La ratios. The difference in Ce/La ratios between the bulk and surface mainly results from the selected subsequent impregnation method, namely La_2_O_2_CO_3_ was first synthesized and then impregnated with Ce precursor solution so that cerium mainly distributed on the surface of the binary oxide.

The morphology and structure of Ce–La binary oxides with different Ce/La ratios were characterized *via* TEM (Fig. S1 in the ESI†). Particles randomly distribute along the surface of La_2_O_2_CO_3_, and some of them are confirmed as CeO_2_. It can be found that small particles have continuous lattice fringes with the oxide hosts, indicating the formation of the solid solution on the interface between the observed small particles and oxide hosts.^37^ Since CeO_2_ diffraction peaks are absent in XRD patterns (Fig. 2), UV-vis is applied to measure the indirect band gap of CeO_2_, which reflects variation tendency in the size of CeO_2_ particles located on the surface of the samples.^49^,^50^ The indirect band gaps of nonoriented polycrystalline CeO_2_ and La_2_O_3_ are 3.19 eV,^49^ and 5.2 eV,^51^ respectively. As shown in Fig. S2 in the ESI,† the indirect band gap of CeO_2_ migrates to a lower value with the increase of Ce/La ratios, indicating the corresponding increment in the size of CeO_2_ particles on the surface of binary oxides. In addition, the variation tendency of the increment remains the same when the Ce/La ratio reaches 0.15 and 0.20. It is reported that the concentration of Ce^3+^ increases with the reduction in CeO_2_ particle size.^47^ Therefore, the obtained results of UV-vis (Fig. S2 in the ESI†) have a similar variation tendency with the results of XPS (Fig. 3).

### *In situ* DRIFTS measurements

**Fig. 4:**
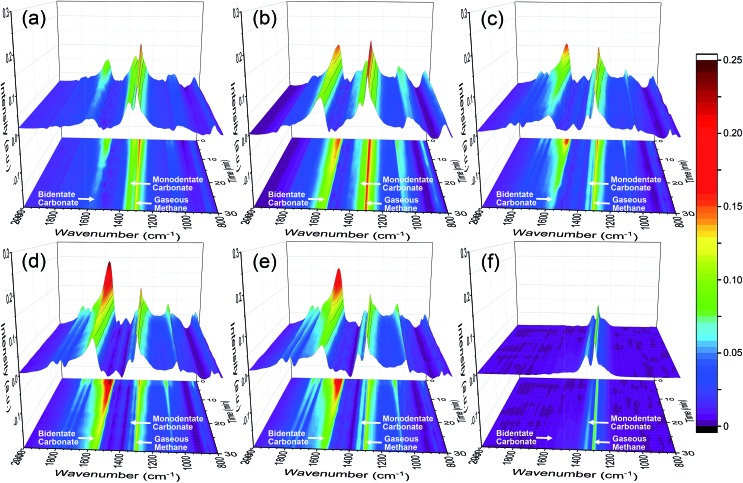
DRIFTS spectra for the series of Ce–La binary oxide during CH_4_ adsorption for 30 min. (a) 0Ce–LOC, (b) 0.05Ce–LOC, (c) 0.10Ce–LOC, (d) 0.15Ce–LOC, (e) 0.20Ce–LOC and (f) CeO_2_.

*In situ* DRIFTS measurements are applied in order to identify surface intermediates present on the Ce–La binary oxide samples from “0 min” to “30 min” (see the definition in the Experimental section), during which coke is formed *via* CH_4_ decomposition and will further react with carbonate formed after CO_2_ adsorption (Fig. 1). *In situ* DRIFTS spectra are shown in Fig. 4, and corresponding contour graphs are inserted in the *x*–*y* planes of Fig. 4 and listed alone in Fig. S3 in the ESI.† The band at 1300 cm^–1^ corresponds to the C–H bending of gaseous methane,^24^,^52^,^53^ and it can be found on all samples. The strong band at 1563 cm^–1^ corresponds to bidentate carbonate with the coexistence of bands at 1300, 1029 and 856 cm^–1^,^15^,^54^,^55^ while the strong band at 1345 cm^–1^ is ascribed to monodentate carbonate with the coexistence of bands at 1428, 1070 and 856 cm^–1^.^15^,^54^,^55^ In addition, bands at 1748 and 1796 cm^–1^ are overtones of C=O stretching vibration and CO_3_^2–^ stretching vibration.^15^

For the series of La–Ce binary oxides, both bidentate carbonate and monodentate carbonate exist on the surface (Fig. 4 and S3 in the ESI†), while for CeO_2_, bidentate carbonate is absent (Fig. 4f and S3f in the ESI†). At “0 min”, the intensity of bidentate carbonate on La–Ce binary oxides increases as the Ce/La ratio increases from 0 to 0.15, reaches the maximum when the Ce/La ratio is 0.15, and finally decreases slightly when the Ce/La ratio reaches 0.20 (Fig. S3 and S4 in the ESI†). Simultaneously, the intensity of monodentate carbonate shows a variation tendency, which is opposite to the intensity of bidentate carbonate. Therefore, it is assumed that Ce addition can influence the CO_2_ adsorption mode on the surface of Ce–La binary oxide and promote the transformation from monodentate carbonate to bidentate carbonate.

**Fig. 5:**
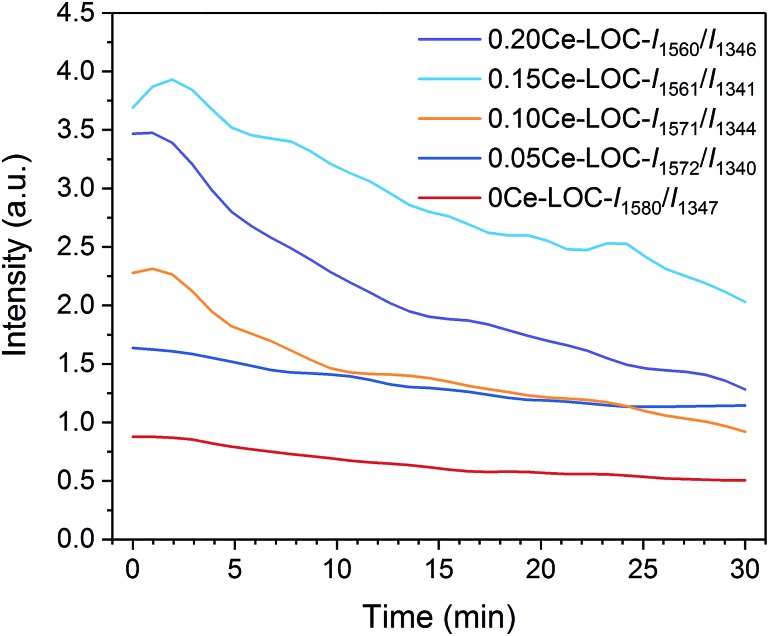
The intensity ratios of bidentate/monodentate carbonate in DRIFTS spectra as a function of time on stream.

When CO_2_ is adsorbed on La_2_O_3_ (30 min prior to “0 min” in Fig. 1), it reacts with La_2_O_3_ and then leads to the formation of La_2_O_2_CO_3_. During the *in situ* DRIFTS measurement (from “0 min” to “30 min” in Fig. 1), CH_4_ can be adsorbed on the surfaces of La_2_O_2_CO_3_ and dissociates to form coke under non-oxidative conditions,^39^,^40^ while coke will react with La_2_O_2_CO_3_ to form La_2_O_3_ and CO.^5^,^8^ For the series of Ce–La binary oxides, the intensity of bidentate carbonate keeps decreasing as time on stream goes by, while the intensity of monodentate carbonate remains stable (Fig. 5 and S3 in the ESI†). Therefore, bidentate carbonate is consumed to react with coke during the introduction of CH_4_. It is concluded that bidentate carbonate is active in the reaction with the coke, while monodentate carbonate is inactive (Fig. 4, S3 and S4 in the ESI†). If we use *I*_B_/*I*_M_ (intensity of ∼1563 cm^–1^/intensity of ∼1345 cm^–1^) to evaluate the ratio of bidentate carbonate to monodentate carbonate, it is found that when the Ce/La ratio equals 0.15, Ce–La binary oxide possesses the highest ratio of *I*_B_/*I*_M_ (Fig. 5). Considering that Ce addition can affect the ratio of *I*_B_/*I*_M_, it is assumed that Ce addition might affect the performance of coke elimination, which will be discussed in the following part.

### Influence on the crystalline phase

XRD patterns of the series of Ce–La binary oxides at “30 min” after reacting with CH_4_ are shown in Fig. 2c and d. The CeO_2_ phase remains absent indicating that cerium is well dispersed. By comparison of the XRD patterns of samples at “0 min” and “30 min”, the intensity of peaks ascribed to II-La_2_O_2_CO_3_ decreases significantly compared with Ia-La_2_O_2_CO_3_. In addition, it is observed that as the Ce/La ratio increases from 0 to 0.15, the decrease in the intensity of II-La_2_O_2_CO_3_ peaks becomes much more prominent. The consumption of II-La_2_O_2_CO_3_ during the CH_4_ adsorption indicates that II-La_2_O_2_CO_3_ is more active than Ia-La_2_O_2_CO_3_ for coke elimination. For the 0.15Ce–LOC sample, the intensity of II-La_2_O_2_CO_3_ is much lower than that of Ia-La_2_O_2_CO_3_ so that Ia-La_2_O_2_CO_3_ eventually becomes the dominant species on the sample, indicating that Ce addition can influence the decomposition of II-La_2_O_2_CO_3_ caused by the reaction with deposited coke.

**Fig. 6:**
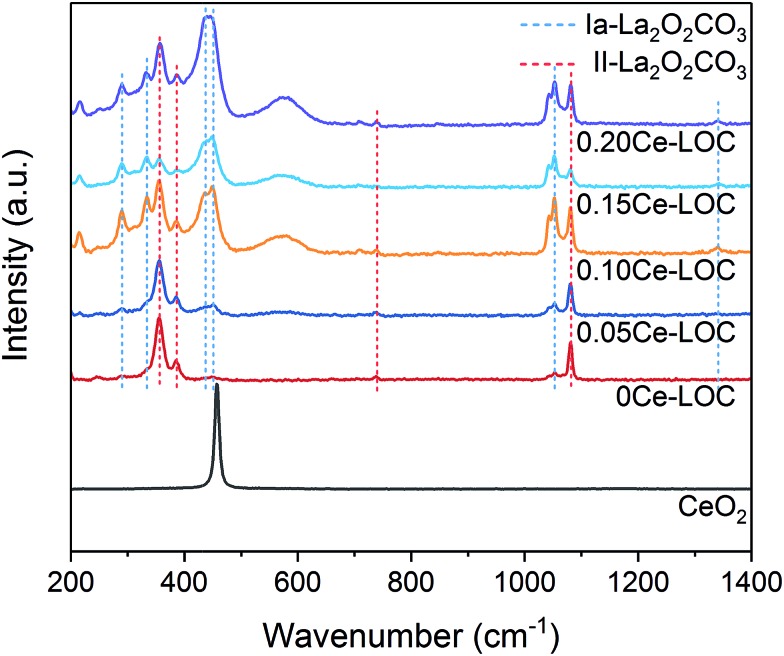
Raman spectra of the series of Ce–La binary oxide upon CH_4_ adsorption for 30 min.

Raman spectra of samples at “30 min” are shown in Fig. 6, which are consistent with the XRD patterns. Peaks at 290, 333, 438, 451, 1052, and 1341 cm^–1^ correspond to Ia-La_2_O_2_CO_3_, while peaks at 355, 385, 740, and 1082 cm^–1^ are assigned to II-La_2_O_2_CO_3_.^13^,^56^,^57^ The intensity ratio of peaks at 355 cm^–1^/451 cm^–1^ can be used as a descriptor to evaluate the dominant surface species. When the Ce/La ratios are 0 and 0.05, the II-La_2_O_2_CO_3_ phase is the dominant surface species and Ia-La_2_O_2_CO_3_ phase is the subordinate. However, when the Ce/La ratio is higher than 0.10, Ia-La_2_O_2_CO_3_ acts as the dominant surface species. For 0.15Ce–LOC, it possesses the lowest intensity ratio of peaks at 355 cm^–1^/451 cm^–1^, indicating that the ratio of II-La_2_O_2_CO_3_/Ia-La_2_O_2_CO_3_ reaches the lowest level. It can be assumed that II-La_2_O_2_CO_3_ formed on the 0.15Ce–LOC sample has the fastest decomposition rate. Two additional peaks at 216 and 577 cm^–1^ are reported to be induced by the dopant,^58^ which verifies the existence of Ce^3+^ in the lattices of the samples.^59^,^60^

DRIFTS results demonstrate that Ce addition is capable of facilitating the formation of bidentate carbonate, which is active for coke elimination. Based on the XRD patterns (Fig. 2) and Raman spectra (Fig. 6), with the decomposition of bidentate carbonate, the ratio of II-La_2_O_2_CO_3_ to Ia-La_2_O_2_CO_3_ will change correspondingly. Spivey *et al.* directly ascribed FTIR bands at 1509 cm^–1^ and 1367 cm^–1^ to II-La_2_O_2_CO_3_ and Ia-La_2_O_2_CO_3_, respectively.^16^ They concluded that only II-La_2_O_2_CO_3_ acts as a reactive species to eliminate coke while Ia-La_2_O_2_CO_3_ merely acts as a spectator species.^16^ However, Kawi *et al.* reported that Ia-La_2_O_2_CO_3_ mainly participated in coke elimination rather than II-La_2_O_2_CO_3_.^61^ Combined with results of XRD patterns, Raman spectra and *in situ* DRIFTS spectra, we could conclude that bidentate carbonate is active for coke elimination and closely related to the II-La_2_O_2_CO_3_ phase while monodentate carbonate is inactive for coke elimination and closely related to the Ia-La_2_O_2_CO_3_ phase.

### Catalytic performance of coke elimination

**Fig. 7:**
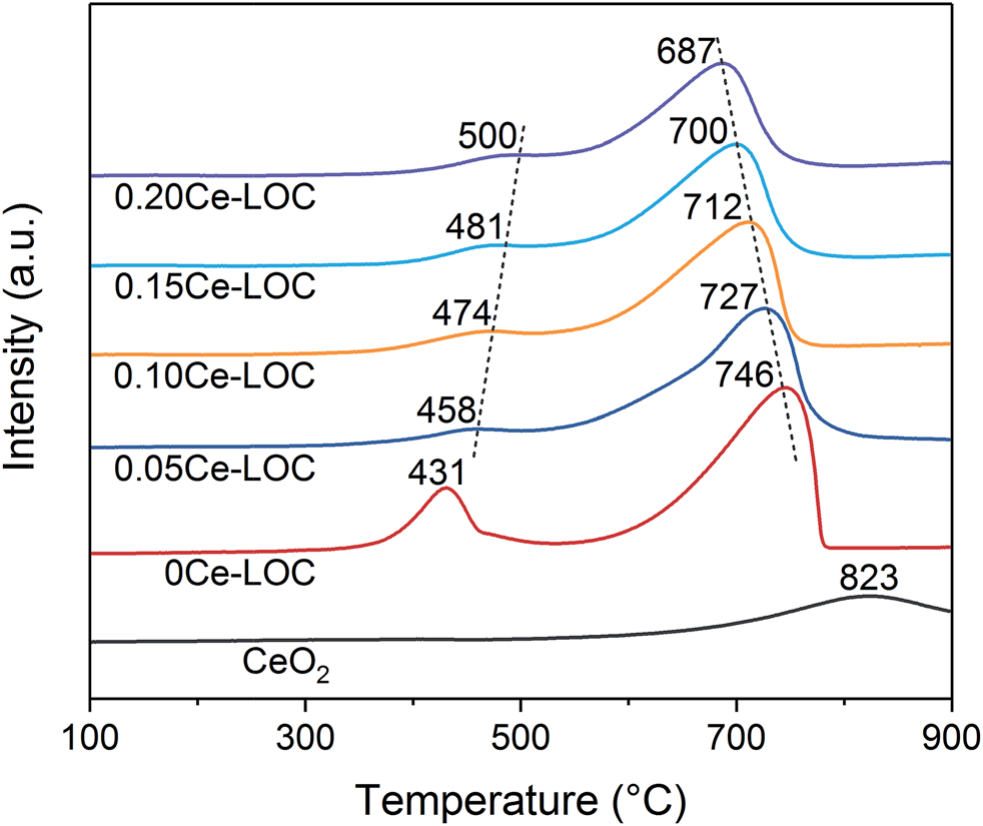
H_2_-TPR profiles of the series of Ce–La binary oxides.

H_2_-TPR was carried out to investigate the decomposition behavior of La_2_O_2_CO_3_ formed on the series of Ce–La binary oxide under a H_2_ atmosphere. The H_2_-TPR profiles are shown in Fig. 7. It should be mentioned that the formation of a reduction peak in H_2_-TPR profiles is due to the reaction of H_2_ and CO_2_ released during the decomposition of La_2_O_2_CO_3_. Herein, peaks at 450–500 °C correspond to the desorption of chemisorbed CO_2_.^9^ With the increase of Ce/La ratios, the desorption temperature of chemisorbed CO_2_ increases to higher temperature. It indicates that Ce addition can strengthen the chemisorption of CO_2_ on the surface of lanthanum species, while peaks at around 700 °C correspond to the decomposition of II-La_2_O_2_CO_3_,^62^ since the phase transformation of Ia-La_2_O_2_CO_3_ to II-La_2_O_2_CO_3_ is complete at around 600 °C.^12^ With the increase of Ce/La ratios, the decomposition temperature of II-La_2_O_2_CO_3_ under the H_2_ atmosphere decreases to a lower temperature, indicating that Ce addition can promote the decomposition of II-La_2_O_2_CO_3_ under the H_2_ atmosphere, which is related to the activity in the reaction of II-La_2_O_2_CO_3_ and coke.

In order to examine the performance of coke elimination, DRM reaction is used as a probe reaction, and then Ni particles are supported on the series of Ce–La binary oxide to produce coke. Ni loadings are fixed at 5 wt% for the series of Ce–La binary oxide to ensure that all catalysts exhibit similar CH_4_ conversions under appropriate reaction conditions.

**Fig. 8:**
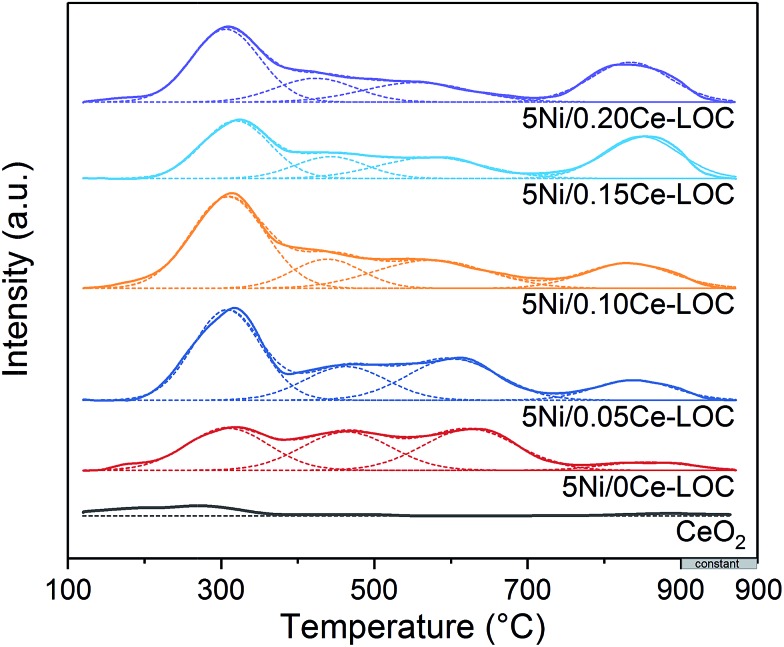
CO_2_-TPD profiles of the series of Ni supported Ce–La binary oxides.

CO_2_-TPD was employed to investigate the basicity of the Ni supported samples (Fig. 8). It has been extensively reported that the basicity of the support is beneficial to coke elimination.^63^ CO_2_ desorption will take place when the reaction temperature (600–650 °C) is higher than the desorption temperature. Thus, basicity with higher desorption temperature should be investigated. CO_2_-TPD profiles show that areas of peaks higher than 700 °C increase with the increment of Ce/La ratio, and reach the maximum when the Ce/La ratio is 0.15, and then decrease slightly when the Ce/La ratio is 0.20. For pure CeO_2_ as a reference, the area of the peak higher than 700 °C is negligible, which indicates that pure CeO_2_ itself has much weaker CO_2_ adsorption compared with Ce–La binary oxide. According to the DRIFTS spectra (Fig. 4, 5 and S3 in the ESI†), for the 0.15Ce–LOC sample, it has the highest intensity of bidentate carbonate during the CH_4_ adsorption. A previous study by Valange *et al.* has shown that bidentate carbonate has higher stability and hence higher basicity compared with monodentate carbonate.^64^ In addition, it has been reported that the decomposition temperature of II-La_2_O_2_CO_3_ is higher than 700 °C.^13^ Therefore, the desorption peaks higher than 700 °C can be ascribed to the decomposition of II-La_2_O_2_CO_3_. It should be mentioned that the atmosphere could affect the decomposition of La_2_O_2_CO_3_,^13^ hence there is a difference in the decomposition temperature of II-La_2_O_2_CO_3_ between CO_2_-TPD and H_2_-TPR (Fig. 7 and 8). For the 0.15Ce–LOC sample, it has the highest amount of bidentate carbonate after CO_2_ adsorption and the highest basicity above 700 °C. It indicates that bidentate carbonate has a close relationship with II-La_2_O_2_CO_3_. Based on these facts, it is assumed that Ce addition can promote the transformation from monodentate carbonate to bidentate carbonate on La_2_O_3_ after CO_2_ adsorption, which will promote the formation of II-La_2_O_2_CO_3_. On the other hand, H_2_-TPR (Fig. 7) profiles show that Ce addition improves the activity of II-La_2_O_2_CO_3_ under the H_2_ atmosphere.

DRM activity tests are applied to test the coke elimination performance of Ce–La binary oxide. GHSV has been adjusted to 60 000 mL h^–1^ g_cat_^–1^ to ensure that different catalysts exhibit similar CH_4_ conversions. Spivey *et al.* reported that when the DRM reaction temperature is 550–650 °C, the variation tendency of coke formation is much more severe since coke originates from both CH_4_ decomposition and Boudouard reaction.^65^ Therefore, the reaction temperature is fixed to 650 °C to increase the coke formation. As shown in Fig. S5 in the ESI,† all the Ni catalysts have CH_4_ conversions at around 44% and exhibit good stability.

**Fig. 9:**
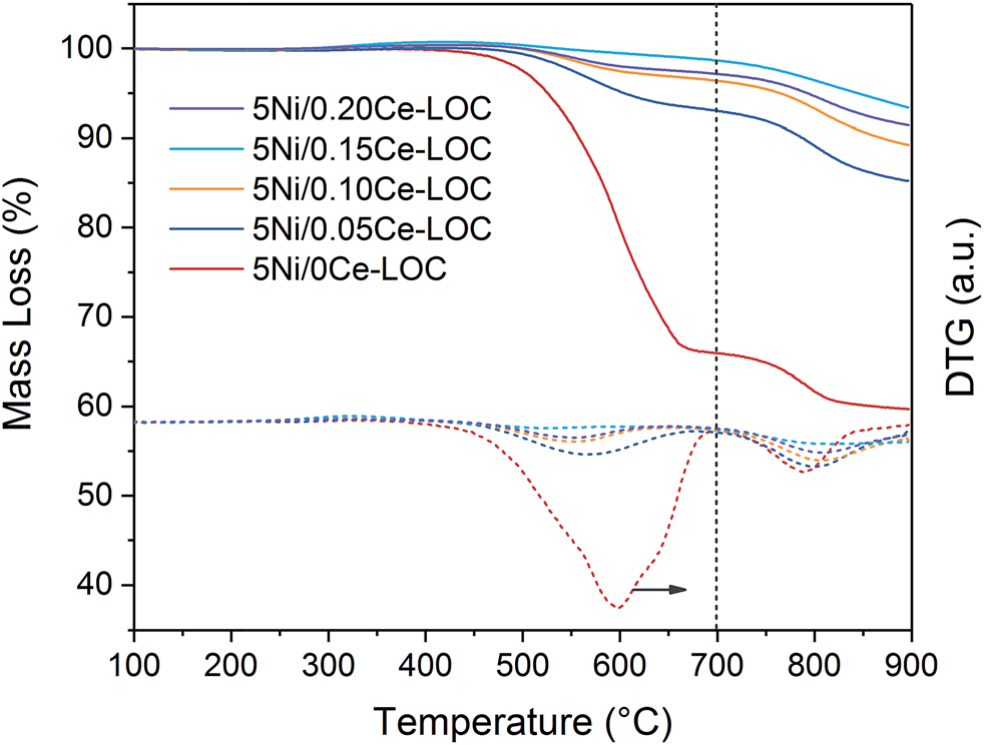
TGA and DTG profiles for the spent catalysts (GHSV = 60 000 mL h^–1^ g_cat_^–1^, 650 °C, time on stream: 50 h).

**Fig. 10:**
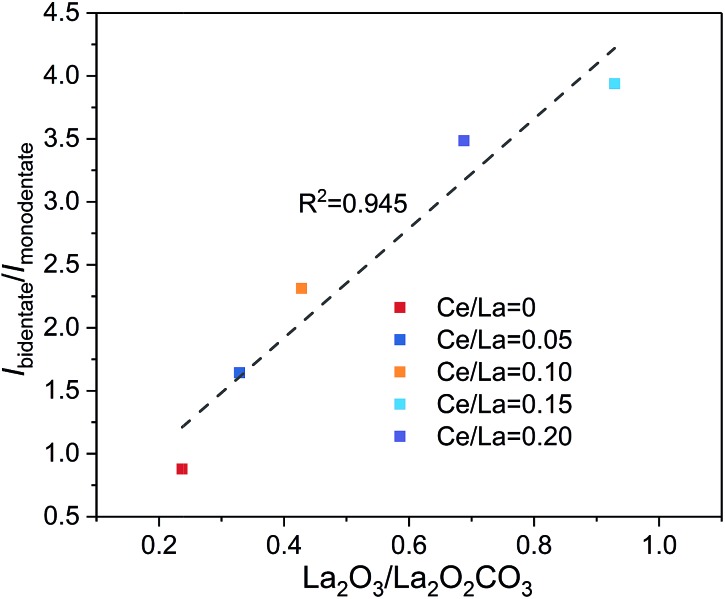
Linear correlation between the molar ratio of La_2_O_3_/La_2_O_2_CO_3_ obtained by TGA profiles and intensity ratio of bidentate/monodentate carbonate obtained by DRIFTS.

TGA profiles are shown in Fig. 9. The mass loss below 700 °C is ascribed to the oxidation of coke and Ni particles. And the mass loss above 700 °C is ascribed to the decomposition of La_2_O_2_CO_3_ to release CO_2_. At the end of TGA, all samples can be regarded as mixtures of NiO, La_2_O_3_ and CeO_2_. According to the Ni loading, specific Ce/La ratio and mass loss obtained by TGA, the content of Ni, CeO_2_, La_2_O_2_CO_3_ and La_2_O_3_ in the spent catalysts can be estimated (Table 2). It should be mentioned that the formation of La_2_O_3_ on the spent catalysts is due to the spontaneous reaction between II-La_2_O_2_CO_3_ and deposited coke. Additionally, since Ia-La_2_O_2_CO_3_ is inactive for coke elimination and spontaneously transforms to II-La_2_O_2_CO_3_ when the temperature is above 600 °C,^12^,^16^ the calculated content of La_2_O_2_CO_3_ (Table 2) is ascribed to the content of Ia-La_2_O_2_CO_3_ which transforms to II-La_2_O_2_CO_3_ during the programmed temperature process. Therefore, we can use the calculated content of La_2_O_3_ and La_2_O_2_CO_3_ in Table 2 to estimate the content of II-La_2_O_2_CO_3_ and Ia-La_2_O_2_CO_3_, respectively. Based on the results of DRIFTS (Fig. 4, 5 and S3 in the ESI†), bidentate carbonate is active for coke elimination while monodentate carbonate is inactive for coke elimination. When correlating the molar ratio of La_2_O_3_/La_2_O_2_CO_3_ in Table 2 with the maximum intensity ratio of bidentate/monodentate carbonate peaks in Fig. 5, a linear relationship is obtained as shown in Fig. 10, which indicates that bidentate carbonate has a close relationship with II-La_2_O_2_CO_3_ while monodentate carbonate is closely related to Ia-La_2_O_2_CO_3_.

**Table 2:** Quantitative analysis of TGA profiles

Sample	Mass loss (%)		Component in the spent catalysts (%)					Molar ratio of La_2_O_3_/La_2_O_2_CO_3_	Amount of coke (g g_cat_^–1^)	Rate of coke formation (μmol g_cat_^–1^·s^–1^)
	<700 °C	>700 °C	Coke	Ni	CeO_2_	La_2_O_2_CO_3_	La_2_O_3_			
5Ni/0Ce–LOC	33.9	6.2	33.9	3.1	0	52.1	10.9	0.237	0.513	0.237
5Ni/0.05Ce–LOC	6.9	7.8	6.9	4.4	4.2	65.5	19.0	0.329	0.074	0.034
5Ni/0.10Ce–LOC	3.9	7.2	3.9	4.5	8.2	60.5	22.8	0.428	0.041	0.019
5Ni/0.15Ce–LOC	2.2	5.3	2.2	4.7	12.1	44.5	36.5	0.929	0.022	0.010
5Ni/0.20Ce–LOC	3.2	5.7	3.2	4.6	15.3	47.9	29.0	0.688	0.033	0.015

In addition, it can be found that the amount of coke decreases with the increase of Ce/La ratio. For 5Ni/0.15Ce–LOC, it has the lowest amount of coke and the highest molar ratio of La_2_O_3_/La_2_O_2_CO_3_ after 50 h DRM reaction, indicating that appropriate Ce addition can promote the formation of II-La_2_O_2_CO_3_ to react with the deposited coke. According to the DTG profiles in Fig. 9, the peak temperature of filamentous coke (∼550 °C) decreases with the increase of Ce/La ratio which reflects the decrease of the graphitic degree of coke,^4^ while the peak temperature of II-La_2_O_2_CO_3_ (>700 °C) exhibits the opposite variation tendency. Based on the above facts, it is concluded that the 0.15Ce–LOC sample shows the best performance for coke elimination.

### DFT calculation of CO_2_ adsorption energy

**Fig. 11:**
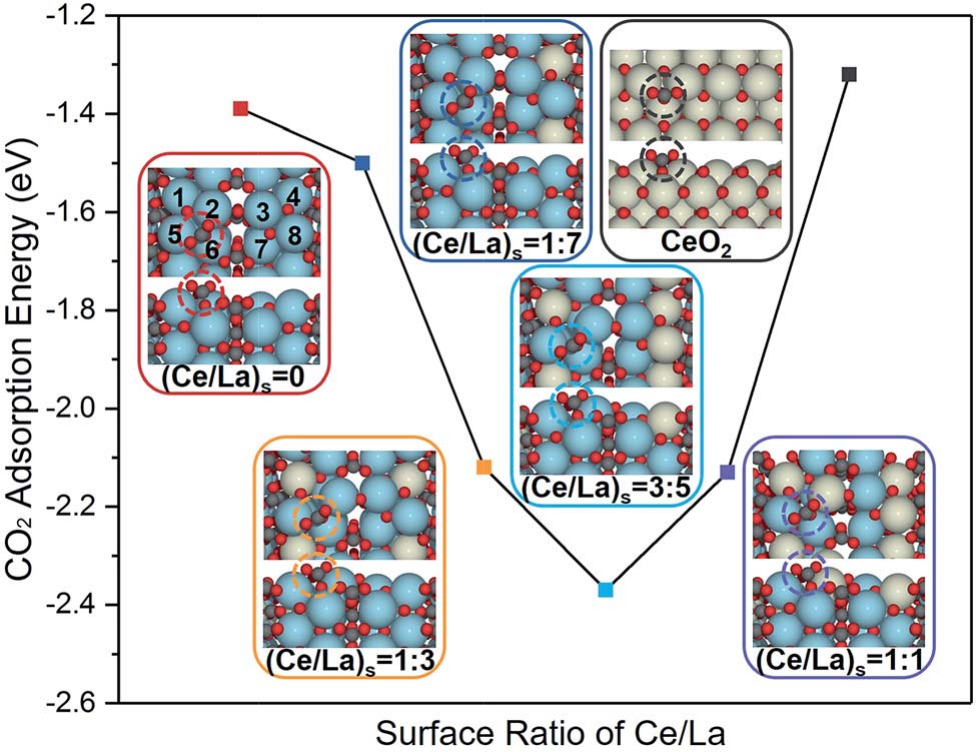
Calculated CO_2_ adsorption energies on the surfaces of Ce–La binary oxides and corresponding top and side views of geometries with CO_2_ adsorbed on the top site of binary oxides. Colors: yellow, Ce; blue, La; grey, C; and red, O.

DFT calculation was applied to illustrate the influence of Ce addition on the most stable CO_2_ adsorption mode on La_2_O_2_CO_3_ (Fig. 11). Ideally, models presuming that Ce atoms distribute along the surface layer were taken into consideration. In this case, a new C–O bond is formed between a CO_2_ molecule with a surface O atom on La_2_O_2_CO_3_, which leads to the formation of carbonate. The calculated frequency of the formed C–O bond is 1517 cm^–1^, which is close to the characteristic frequency of bidentate carbonate (1560 cm^–1^) collected by *in situ* DRITFS (Fig. 4). In addition, the calculated CO_2_ adsorption energy for pure II-La_2_O_2_CO_3_ ((Ce/La)_s_ is equal to 0, the subscript s denotes surface) is –1.39 eV, which is a negative value since it is an exothermic adsorption process.

When the ratio of (Ce/La)_s_ is equal to 1 : 7, there are eight possible sites for a Ce atom to replace a La atom within our selected unit cell (Fig. S6 in the ESI†). DFT calculations predict that the most stable CO_2_ adsorption mode takes place when the La atom at site 4 (see the definition in Fig. 11) is replaced by a Ce atom and the calculated CO_2_ adsorption energy is –1.50 eV. For samples with higher Ce content, the selected model was based on the most stable structure of samples with lower Ce contents. For example, when the ratio of (Ce/La)_s_ is equal to 1 : 3, one Ce atom is fixed at the fourth site on the basis of the obtained calculation (Fig. S7 in the ESI†). In this case, the most stable CO_2_ adsorption mode takes place when La atoms located at sites 1 and 4 are replaced by Ce atoms and the calculated adsorption energy is –2.12 eV. Following the same procedure, when the ratio of (Ce/La)_s_ increases to 3 : 5 and 1 : 1, the strongest adsorption energy of CO_2_ over Ce–La binary oxides is –2.37 eV (Fig. S8 in the ESI†) and –2.13 eV (Fig. S9 in the ESI†), respectively. Meanwhile, twenty extra models with randomly distributed Ce structures were tested, which did not follow the mentioned procedure. The CO_2_ binding over all these randomly generated models is less stable than the ones discussed above (Table S1 in the ESI†). Additionally, the calculated CO_2_ adsorption energy for pure CeO_2_ is –1.32 eV, which is weaker than that of La_2_O_2_CO_3_ and other Ce–La binary oxides. It indicates that the intensity of CO_2_ adsorption on ceria is much weaker than that of La_2_O_2_CO_3_ and other Ce–La binary oxides, which is responsible for the absence of bidentate carbonate on CeO_2_ as shown in Fig. 4.

According to the results of DFT calculation (Fig. 11), with the increase of (Ce/La)_s_ ratios, the CO_2_ adsorption energy gradually becomes lower and reaches the lowest value when (Ce/La)_s_ is equal to 3 : 5, and then the CO_2_ adsorption energy weakens. The variation tendency of CO_2_ adsorption energy with the increase of (Ce/La)_s_ ratios (Fig. 11) is consistent with the variation tendency for the peak intensity of bidentate carbonate on Ce–La binary oxides (Fig. 4). As the CO_2_ adsorption energy becomes lower, CO_2_ adsorption on binary oxide is strengthened, and the formed carbonate is expected to have better stability. Therefore, Ce addition can affect CO_2_ adsorption energy for Ce–La binary oxide and the type of carbonate formed after CO_2_ adsorption. Ce–La binary oxide with the optimal Ce/La ratio exhibits the highest intensity of bidentate carbonate (Fig. 4), and hence has the highest basicity above 700 °C (Fig. 8) and shows the best coke elimination performance (Fig. 9).

## Conclusion

We have investigated the role of Ce addition in CO_2_ adsorption and activation over lanthanum species. Based on results of *in situ* DRIFTS spectra, DFT calculations and other experimental characterizations, it is concluded that Ce addition can promote the formation of bidentate carbonate on Ce–La binary oxide *via* tuning CO_2_ adsorption energy. With the increase of Ce/La ratio from 0 to 0.20, Ce addition facilitates transformation from monodentate carbonate to bidentate carbonate on Ce–La binary oxides. Bidentate carbonate is verified to be active in the reaction with deposited coke, while monodentate carbonate is inactive for coke elimination. With the consumption of bidentate carbonate, the variation of the intensity ratio of bidentate/monodentate carbonate can affect the ratio of II-La_2_O_2_CO_3_/Ia-La_2_O_2_CO_3_. Bidentate carbonate is verified to be closely related to II-La_2_O_2_CO_3_ and monodentate carbonate has a close relationship with Ia-La_2_O_2_CO_3_. When the Ce/La ratio is 0.15, the corresponding nickel catalyst has the highest intensity ratio of bidentate/monodentate carbonate and the highest ratio of II-La_2_O_2_CO_3_/Ia-La_2_O_2_CO_3_, which exhibits the highest basicity above 700 °C and the best performance of coke elimination after 50 h DRM reaction.

## Experimental section

### Catalyst preparation

Analytical grade La(NO_3_)_3_·6H_2_O, Ce(NO_3_)_3_·6H_2_O, Ni(NO_3_)_2_·6H_2_O and CeO_2_ powder were obtained from Aladdin Industrial Corporation (Shanghai, China). Analytical grade aqueous ammonia (25 wt%) and anhydrous ethanol (99.8 wt%) were obtained from Guangfu Fine Chemical Research Institute (Tianjin, China). Guaranteed grade urea was supplied by Kermel Chemical Reagent (Tianjin, China). De-ionized water (18.0 MΩ) was prepared using an Ulupure water purifier machine (Chengdu, China).

The synthesis route of La_2_O_2_CO_3_ is described as follows. 2.6 grams of La(NO_3_)_3_·6H_2_O and 7.2 grams of urea were separately dissolved in de-ionized water. Once dissolved, the two solutions were mixed with constant stirring; the concentrations of La^3+^ and urea in the mixture were 0.015 mol L^–1^ and 0.30 mol L^–1^, respectively. Then aqueous ammonia was added into the mixture to adjust the pH to 8.5. A white suspension was obtained after heating in a water bath at 90 °C for 3 h with constant stirring, followed by naturally cooling to room temperature. Afterwards, the white suspension was centrifuged and washed with absolute ethanol three times. La_2_O_2_CO_3_ was finally prepared upon drying at 80 °C overnight and calcination at 500 °C for 2 h.

A series of Ce–La binary oxides were prepared by a wet impregnation method. The stoichiometric Ce/La ratio was chosen as 0, 0.05, 0.10, 0.15, and 0.20, respectively. The prepared La_2_O_2_CO_3_ was impregnated with an aqueous solution containing a specified amount of Ce(NO_3_)_2_·6H_2_O. Upon stirring at 80 °C for 3 h, vacuum evaporation was carried out to remove the solvent. Then the sample was dried overnight, and ground and calcined at 600 °C for 2 h. The prepared Ce–La binary oxide was marked as “*x*Ce–LOC”, where LOC denotes the prepared La_2_O_2_CO_3_ and *x* denotes the specific stoichiometric Ce/La ratio.

A series of Ni catalysts supported on the prepared Ce–La binary oxide were synthesized by a similar procedure to that described above. For the subsequent wet impregnation method the Ni loading was fixed at 5 wt% on the basis of reduction conditions. When the sample was impregnated with the Ni precursor and dried overnight, it was ground and calcined at 650 °C for 2 h. After grinding to 20–40 mesh, the sample was reduced at 650 °C under a H_2_ atmosphere (H_2_/N_2_ = 1 : 3, 40 mL min^–1^) for 60 min. The prepared catalyst was named 5Ni/*x*Ce–LOC, where LOC denotes the prepared La_2_O_2_CO_3_ and *x* denotes the specific Ce/La ratio.

### Characterization

Textural properties of catalysts were measured through nitrogen adsorption–desorption at –196 °C using a Micromeritics Tristar 3000 analyzer. All samples were degassed at 300 °C for 3 h prior to the tests. The specific surface areas were calculated on the basis of the N_2_ isotherms and the Brunauer–Emmett–Teller (BET) method. Combined with the Barrett–Joyner–Halenda (BJH) method and the desorption branches of the N_2_ isotherms, cumulative volumes of pores were obtained.

Elemental contents of the prepared catalysts were examined by inductively coupled plasma optical emission spectroscopy (ICP-OES) (VISTA-MPX, Varian) at a high frequency emission power of 1.5 kW and a plasma airflow of 15.0 L min^–1^ (*λ*_Ni_ = 231.60 nm, *λ*_La_ = 379.48 nm, *λ*_Ce_ = 413.76 nm). Prior to measurements, samples were dissolved in a mixture of nitric acid and H_2_O_2_ to ensure that the concentrations of the measured elements are close to the concentrations of the prepared standard solutions.

XRD patterns were examined through a Rigaku D/max-2500 diffractometer equipped with graphite filtered Cu Kα radiation (*λ* = 1.54056 Å), and 2*θ* values range from 20° to 80°. The mean crystalline size of Ni particles was calculated by Scherrer's equation according to the diffraction peaks of Ni (111) facets.

H_2_-TPR experiment was applied to analyze the reduction behavior of the catalysts with the aid of a chemisorption apparatus (Micromeritics AutoChem II 2920). 100 mg of the sample was pretreated at 300 °C for 1 h with an Ar stream (30 mL min^–1^) to remove moisture and impurities. After cooling to 50 °C, the system was exposed to a 10 vol% H_2_/Ar stream (30 mL min^–1^) to reduce the sample. Subsequently, the temperature of the system was programmed to rise linearly from 100 °C to 900 °C with a rate of 10 °C min^–1^, during which variation of the signal of the thermal conductivity detector (TCD) was recorded.

CO_2_-TPD analysis was applied to investigate the basicity of the catalyst by utilizing the same chemisorption apparatus (Micromeritics AutoChem II 2920). 100 mg of the sample was prereduced at 750 °C with a 10 vol% H_2_/Ar stream (50 mL min^–1^) for 30 min to completely decompose existing La_2_O_2_CO_3_ on samples before CO_2_ adsorption. After cooling to 60 °C, the system was exposed to a stream of CO_2_ gas (50 mL min^–1^) to carry out CO_2_ adsorption for 30 min. Next, the system was exposed to a He stream (30 mL min^–1^) and the temperature was programmed to increase to 120 °C for the removal of residual CO_2_ in the stream. Once the signal of TCD reached a stable state, the temperature of the system was programmed to increase from 120 °C to 900 °C with a ramping rate of 10 °C min^–1^ and at the same time the system starts to keep record of the TCD signal. An isothermal period lasting for 8 minutes at 900 °C was set to ensure that the adsorbed CO_2_ was totally desorbed.

A TEM instrument (FEI Tecnai G2 F20) was applied to investigate the morphology and structure of catalysts, and the working voltage was 100 kV. After the sample powder was dispersed in absolute ethanol *via* ultrasonication, the obtained suspension was dripped onto a copper grid-supported transparent carbon foil and dried in air for characterization.

XPS analysis of the catalysts was carried out on a Perkin-Elmer PHI 1600 ESCA system equipped with an Al KR X-ray source (*E* = 1486.6 eV). Spectra were operated at a pass energy of 187.85 eV. The binding energy (B.E.) scale was measured on the basis of carbon contamination utilizing C 1s peak centered at 285 eV. In addition, core peaks were obtained using a nonlinear Shirley-type background. Besides, quantification of surface elemental composition was carried out according to Scofield's relative sensitivity factors.^66^

Properties of the coke deposited on the spent catalysts were characterized by utilizing a TGA system (STA449F3, NETZSCH Corp.). The TGA experiment was conducted in an air stream (50 mL min^–1^), and the temperature was programmed to rise from room temperature to 900 °C with a heating rate of 10 °C min^–1^. The amount of coke deposition, II-La_2_O_2_CO_3_ accumulation and oxidation of Ni particles were calculated according to the mass losses in TGA profiles.

A Raman spectrometer (Renishaw inVia Reex) was employed to record Raman spectra under ambient conditions, which was equipped with a 532 nm Ar-ion laser beam as the excitation source. Each sample was examined more than three times at different positions.

UV-visible reflectance spectra were collected on a SHIMADZU UV-2550 spectrophotometer using a pressed disc of the sample. Kubelka–Munk transformed diffuse reflectance spectra (DRS) of all samples were measured with BaSO_4_ powder as a reference.

### *In situ* DRIFTS measurements

*In situ* DRIFTS experiments were performed on a ThermoFisher Nicolet IS50 spectrometer, which was equipped with a Harrick Scientific diffuse reflection accessory and a mercury–cadmium–telluride (MCT) detector. The scheme of the experimental process is shown in Fig. 1. The Ce–La binary oxide samples were placed in the cell of the DRIFTS apparatus and reduced at 600 °C under 30 vol% H_2_/Ar stream (30 mL min^–1^). And then, the gas stream was switched from H_2_/Ar stream to Ar stream (30 mL min^–1^) in order to remove hydrogen in the gas stream. Subsequently, the baseline of DRIFTS was collected continuously until the obtained baseline spectra remained stable. Afterwards, the gas stream was switched to 10 vol% CO_2_/Ar stream (30 mL min^–1^) to carry out CO_2_ adsorption for 30 min, after which the CO_2_ adsorption was saturated. The moment at the end of CO_2_ adsorption was marked as “0 min”. Subsequently, the gas stream was switched from 10 vol% CO_2_/Ar stream to 10 vol% CH_4_/Ar stream (30 mL min^–1^) in order to carry out CH_4_ adsorption for 30 min. The moment at the end of CH_4_ adsorption was marked as “30 min”. *In situ* DRIFTS measurements were carried out from “0 min” to “30 min”. Since DRIFTS spectra remained stable after “30 min”, it could be regarded that the reaction took place completely. Additionally, when it is “0 min” or “30 min”, subsequent operations could be skipped and the temperature would decrease from 600 °C to room temperature. When the cell was cooled to room temperature, samples could be taken out to carry out *ex situ* characterizations.

### Periodic DFT calculations

Periodic DFT calculations were carried out with the assistance of Vienna *ab initio* Simulation Package (VASP).^67^ The calculations employed the generalized-gradient approximation (GGA) in the form of the Perdew–Burke–Ernzerhof (PBE) exchange–correlation functional.^68^ A Hubbard U value was added to the PBE functional (DFT + U), which is chosen to improve the quality of the DFT calculations in dealing with oxides having narrow f or d bands.^69^ The interactions between the atomic cores and electrons were described by the projector augmented wave (PAW) method.^70^ The valence wave functions were expanded using plane-wave with a cutoff energy of 400 eV. A 4 × 2 cell was used for La_2_O_2_CO_3_ (110) and CeO_2_ (110) surfaces, and a 3 × 1 × 1 *k*-point mesh was used for the Brillouin zone integration. The slab was three layers thick and separated by 15 Å of vaccum. The top two layers of the slab were allowed to relax, while the bottom one layer was kept fixed. For La, a value of *U*_eff_ = 7.5 eV was used, which was calculated self-consistently by Metiu *et al.*^19^*U*_eff_ = 4.5 eV was employed for Ce, which was reported by Fabris *et al.*^71^

The adsorption energy of adsorbates, *E*_ads_, is defined as follows:

2 *E*_ads_ = *E*_total_ – *E*_gas_ – *E*_slab_,

where *E*_total_ is the total energy of the system after adsorption, *E*_gas_ is the energy of the adsorbate in the gas phase, and *E*_slab_ is the energy of the clean slab. Thus, a negative value means an exothermic adsorption process.

### Coke elimination performance test

Catalytic activity tests were carried out in a quartz fixed-bed tubular reactor (*Φ* 8 × 44 mm) under atmospheric pressure. Prior to the test, the catalyst sample (100 mg, 20–40 mesh) was evenly mixed with 1 mL of quartz particles and then the mixture was loaded inside the reactor. Prior to the test, the catalysts were reduced at 650 °C under a 25 vol% H_2_/N_2_ stream (40 mL min^–1^) for an hour. The flow rate of the feed gas was set at 100 mL min^–1^ (GHSV = 60 000 mL h^–1^ g_cat_^–1^, CH_4_ : CO_2_ : N_2_ = 20 : 20 : 60 mL min^–1^) to ensure that the activities of the catalysts are close to each other to simplify the comparison of coke deposition. Here, the mentioned GHSV is based on a total flow. The concentrations of gas species including CH_4_, CO_2_, H_2_, CO, and N_2_ were measured online with the assistance of a gas chromatograph (GC2060, Shanghai Ruimin Instrument). Helium was used as the carrier gas. The GC was equipped with a TCD and two columns including a TDX-01 column followed by a 5 A molecular sieve column. Activity test was performed at 650 °C for 50 h. Conversions of CH_4_ and CO_2_ (*X*_CH_4__ and *X*_CO_2__), selectivities to H_2_ and CO (*S*_H_2__ and *S*_CO_), and the H_2_/CO ratio are defined as follows:
3

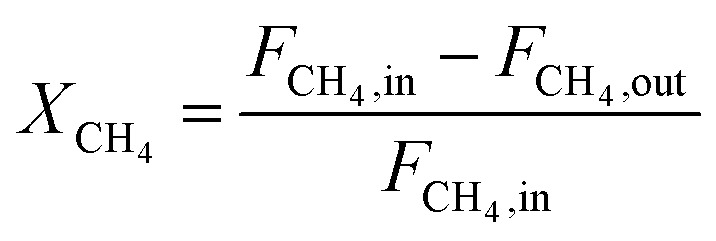



4

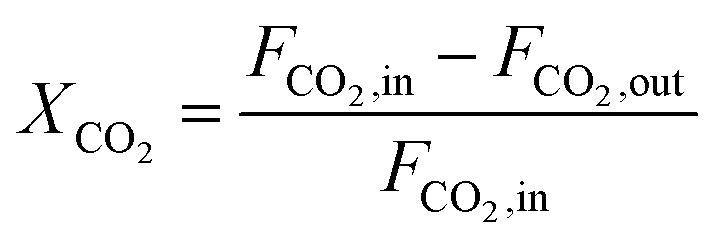



5

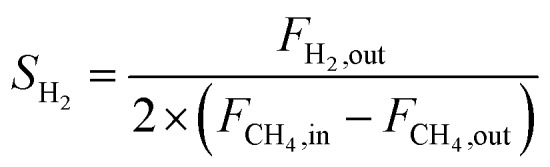



6

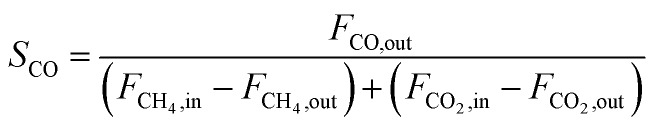



7

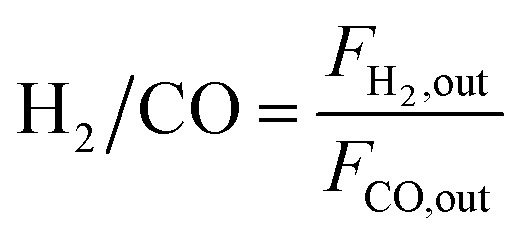




## Conflicts of interest

There are no conflicts to declare.

## Supplementary Material

Supplementary information
