# Supplementary material for: On the role of Ce in CO2 adsorption and activation over lanthanum species
Source: Chem Sci. 2018 Feb 23;9(14):3426–37. doi: 10.1039/c8sc00203g (PMC5932599; doi:10.1039/c8sc00203g)
Supplement: Supplementary file 1 [file SC-009-C8SC00203G-s001.pdf]

Electronic Supplementary Material (ESI) for Chemical Science.
This journal is © The Royal Society of Chemistry 2018

# Supporting Information

# On the Role of Ce towards $CO_2$ Adsorption and Activation over Lanthanum Species

Xinyu Li,[ab1] Zhi-Jian Zhao,[ab1] Liang Zeng,[ab] Jiubing Zhao,[ab] Hao Tian,[ab]

Sai Chen,[ab] Kang Li,[ab] Sier Sang,[ab] and Jinlong Gong*[ab]

[a] *Key Laboratory for Green Chemical Technology of Ministry of Education, School of Chemical Engineering and Technology, Tianjin University, Tianjin 300072, China.*

[b] *Collaborative Innovation Center of Chemical Science and Engineering (Tianjin), Tianjin 300072, China.*

* Corresponding author. Fax: +86-22-87401818; Email address: jlgong@tju.edu.cn.

[1] These authors contributed equally to this work.

# Table of Contents

**Figure S1.** HR-TEM images of the series of Ce-La mixed oxide after $CO_2$ adsorption for 30 min3

**Figure S2.** Plots of $(ahv)^2$ vs photon energy for the series of Ce-La mixed oxide....................... 4

**Figure S3.** Contour graphs of DRIFTS spectra for the series of Ce-La mixed oxide during $CH_4$ adsorption for 30 min.......................................................................................................... 5

**Figure S4.** The intensity of peaks in DRIFTS spectra as a function of time on stream............... 6

**Figure S5.** Activity test of the catalysts .................................................................................. 7

**Figure S6.** Possible models for 1Ce-doping and corresponding calculated $CO_2$ adsorption energies ..........................................................................................................................................8

**Figure S7.** Possible models for 2Ce- doping and corresponding calculated $CO_2$ adsorption energies.......................................................................................................................... 9

**Figure S8.** Possible models for 3Ce- doping and corresponding calculated $CO_2$ adsorption energies.......................................................................................................................... 10

**Figure S9.** Possible models for 4Ce- doping and corresponding calculated $CO_2$ adsorption energies.......................................................................................................................... 11

**Table S1.** Calculated $CO_2$ adsorption energies with randomly distributed Ce structures .......... 12

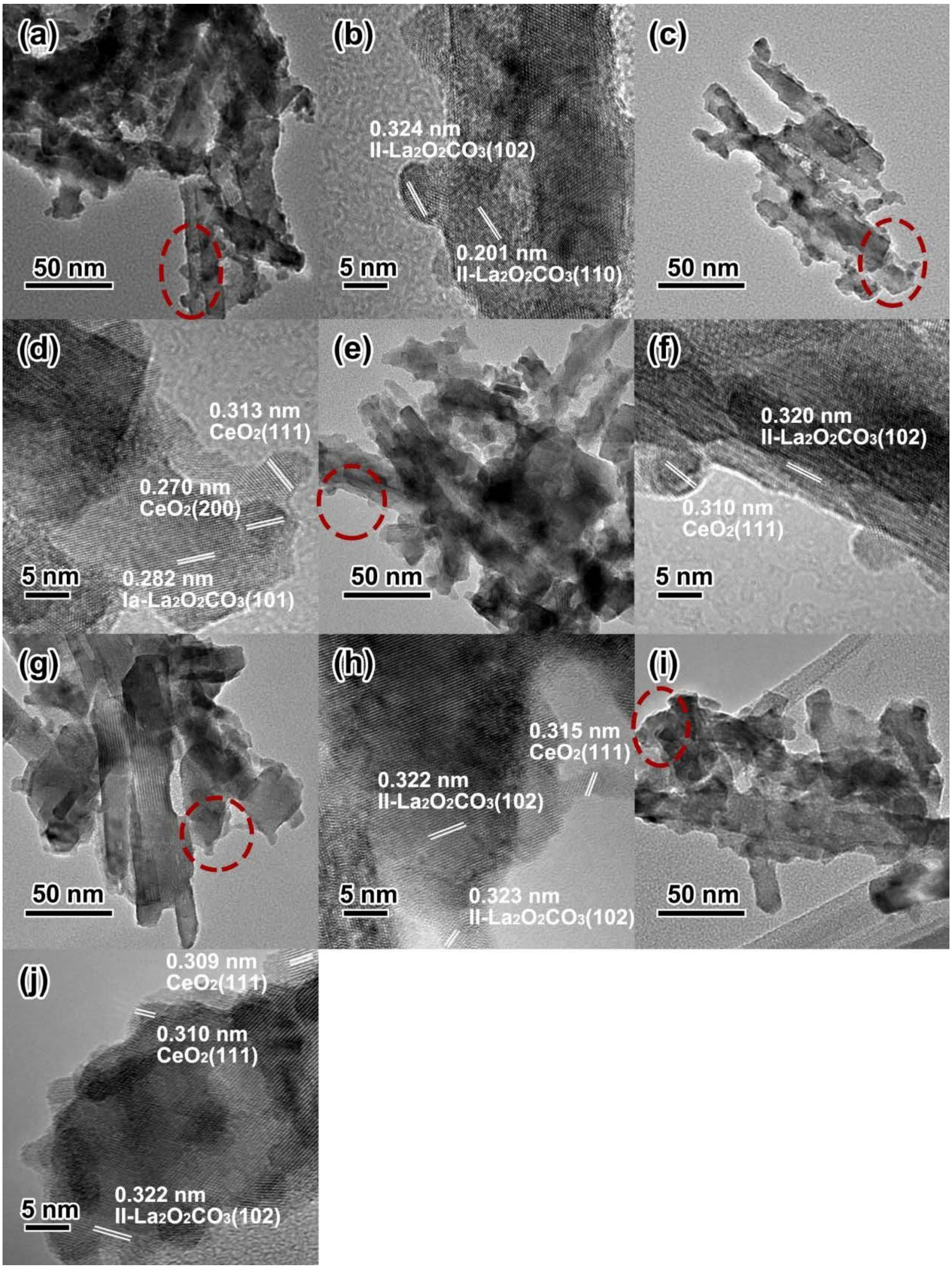


**Figure S1.** HR-TEM images of the series of Ce-La mixed oxide after $CO_2$ adsorption for 30 min. (a, b) $La_2O_2CO_3$, (c, d) 0.05Ce-LOC, (e, f) 0.10Ce-LOC, (g, h) 0.15Ce-LOC, (i, j) 0.20Ce-LOC.

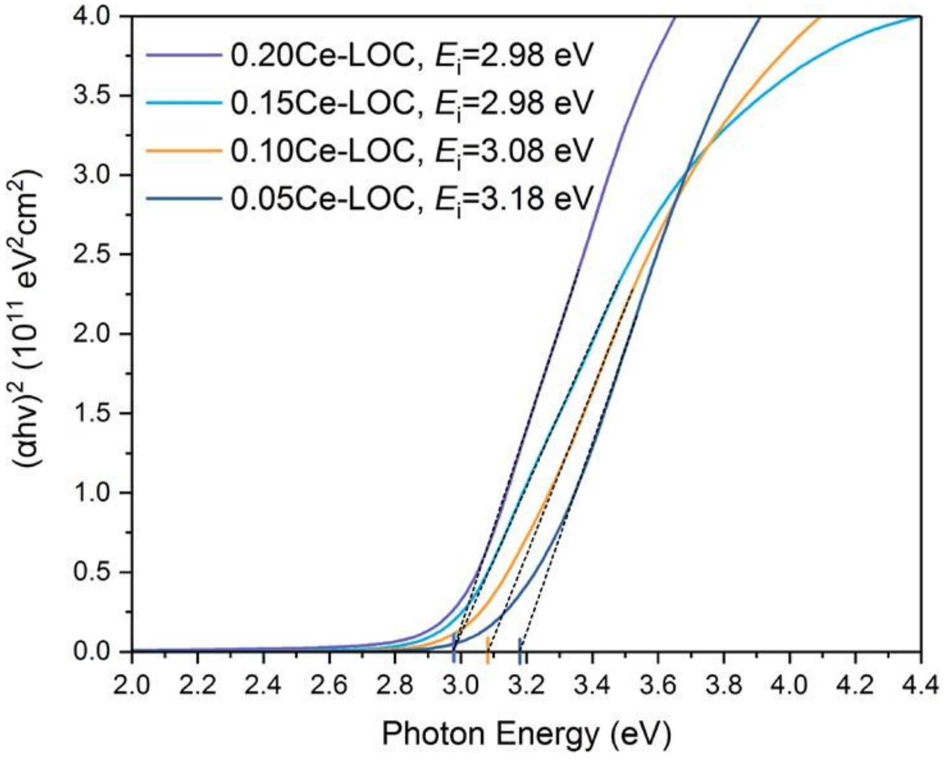


**Figure S2.** Plots of $(ahv)^2$ vs photon energy for the series of Ce-La mixed oxides.

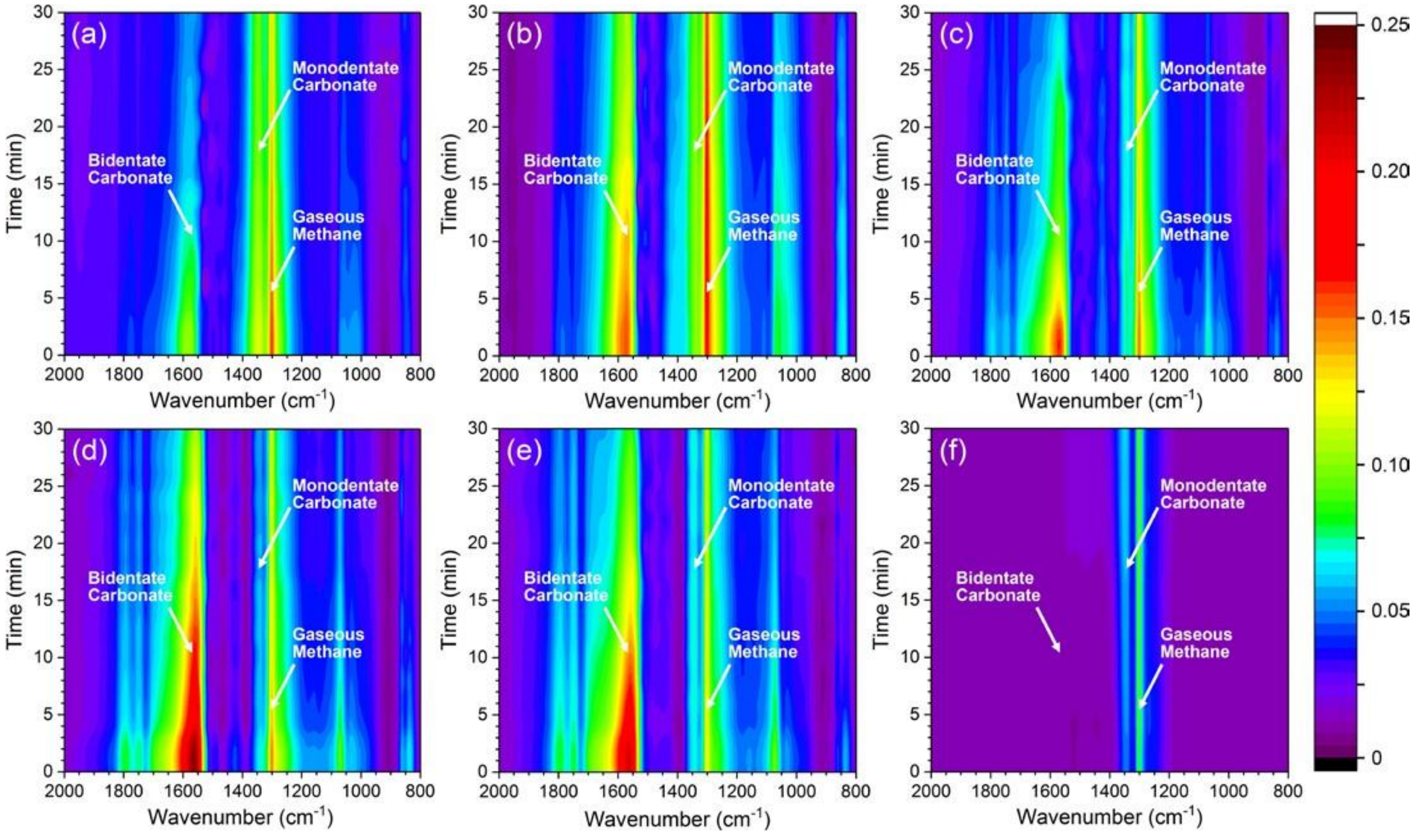


**Figure S3.** Contour graphs of DRIFTS spectra for the series of Ce-La mixed oxide during $CH_4$ adsorption for 30 min. (a) 0Ce-LOC, (b) 0.05Ce-LOC, (c) 0.10Ce-LOC, (d) 0.15Ce-LOC, (e) 0.20Ce-LOC and (f) $CeO_2$.

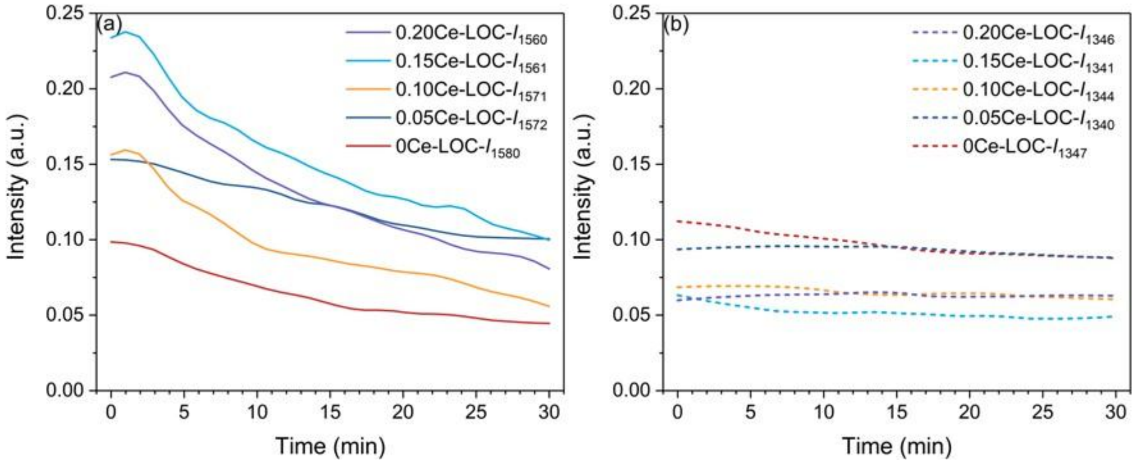


**Figure S4.** The intensity of peaks in DRIFTS spectra as a function of time on stream. (a) Bidentate carbonate, (b) Monodentate carbonate.

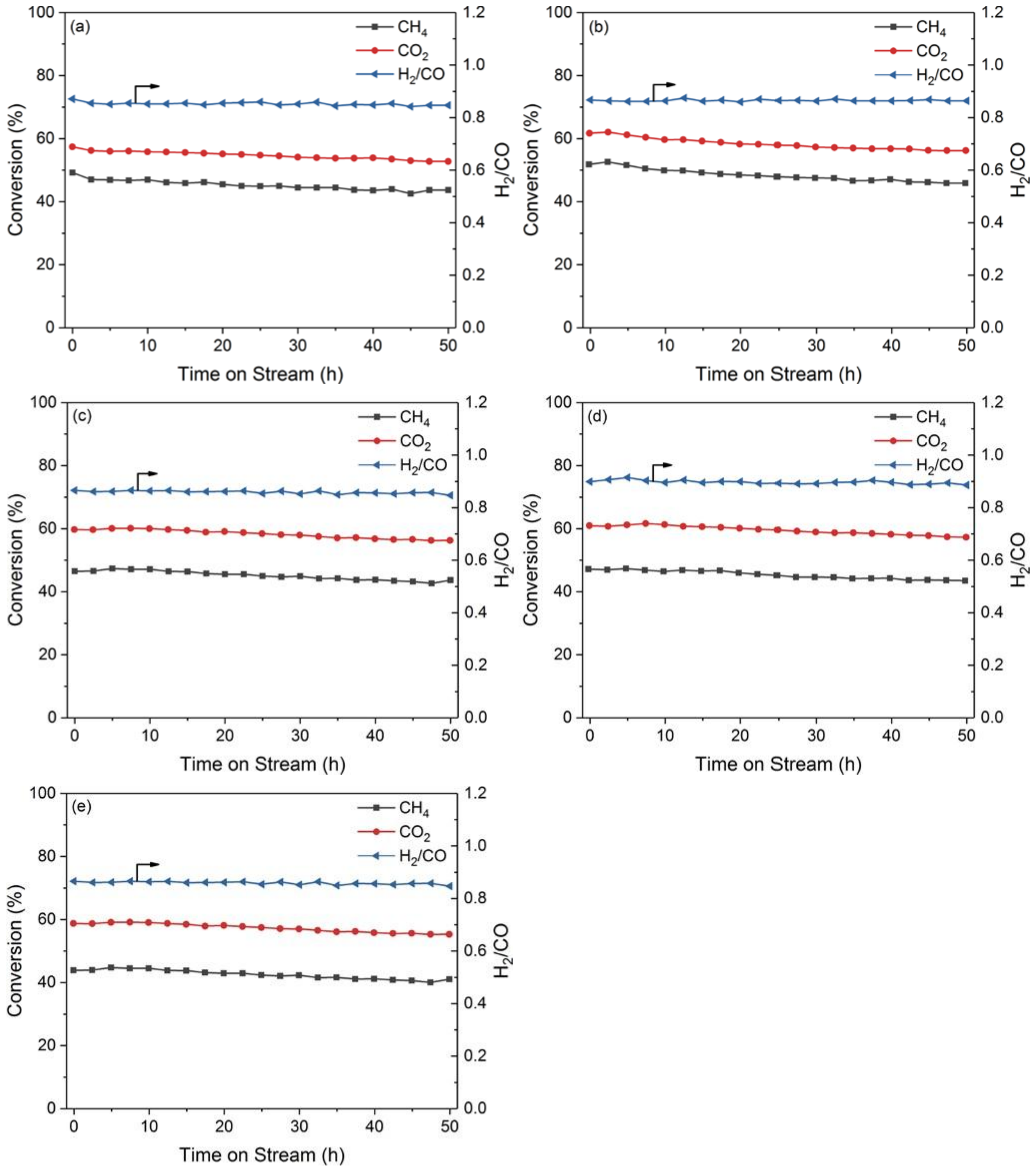


**Figure S5.** Activity test of the catalysts. $CH_4$ and $CO_2$ conversions as a function of time on stream over the catalysts including relevant $H_2$/CO ratio. (a) 5Ni/0Ce-LOC, (b) 5Ni/0.05Ce-LOC, (c) 5Ni/0.10Ce-LOC, (d) 5Ni/0.15Ce-LOC, and (e) 5Ni/0.20Ce-LOC.Reaction conditions: $CH_4/CO_2/N_2$ = 20/20/60, GHSV = 60,000 mL·$h^{-1}$·$g_{cat}^{-1}$, 650 °C, 1 atm.

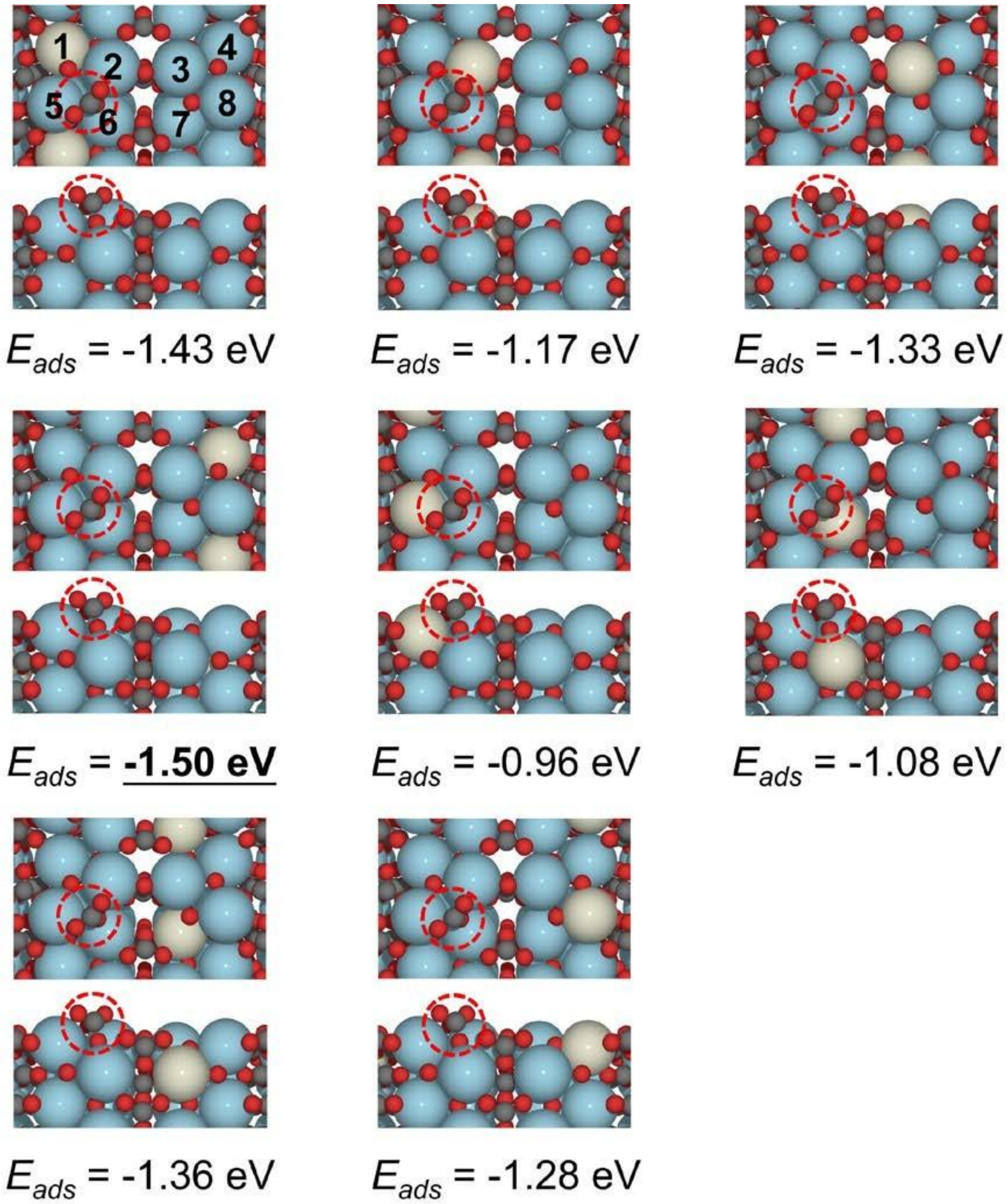


**Figure S6.** Possible models for 1Ce-doping and corresponding calculated $CO_2$ adsorption energies.

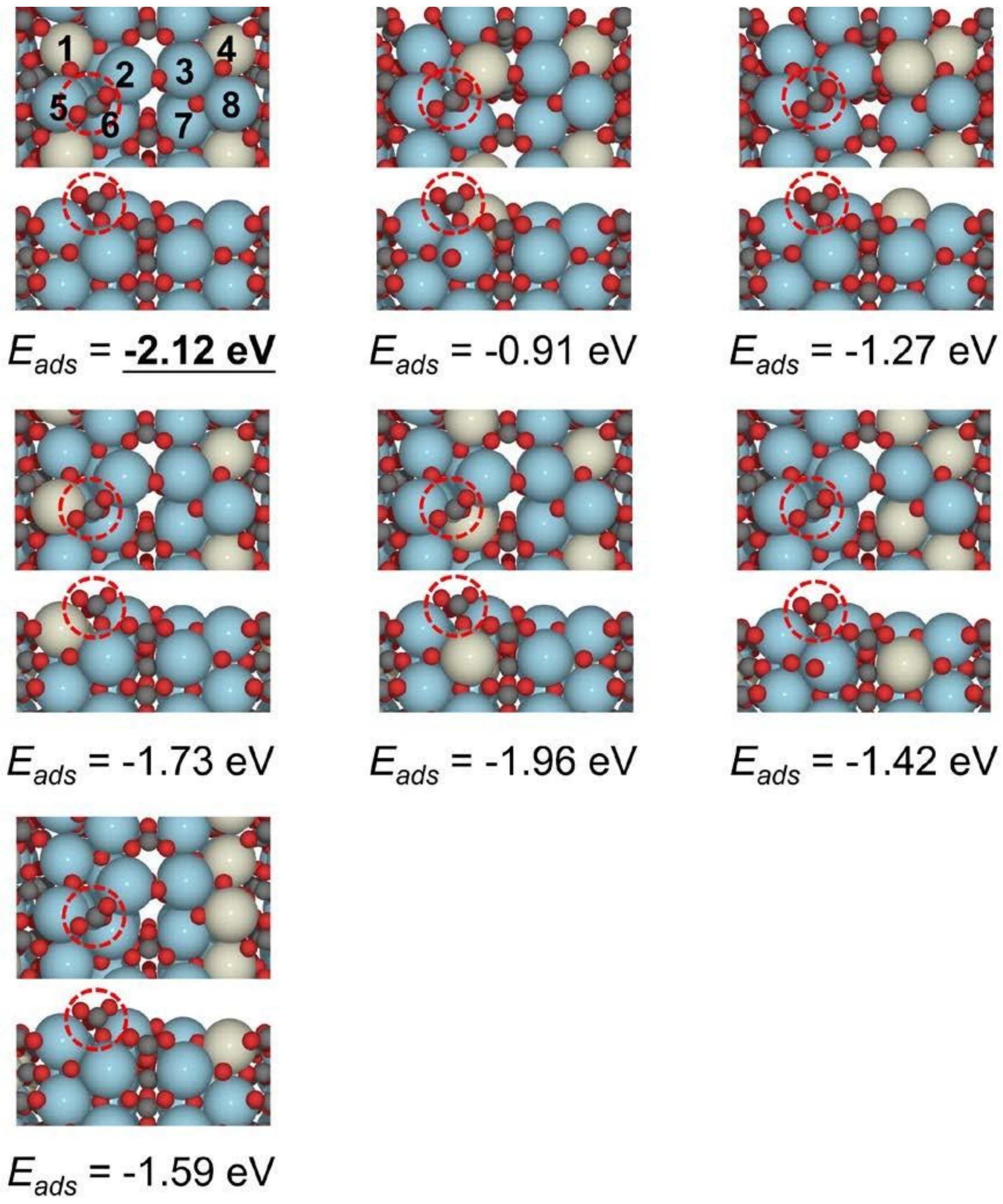


**Figure S7.** Possible models for 2Ce-doping and corresponding calculated $CO_2$ adsorption energies.

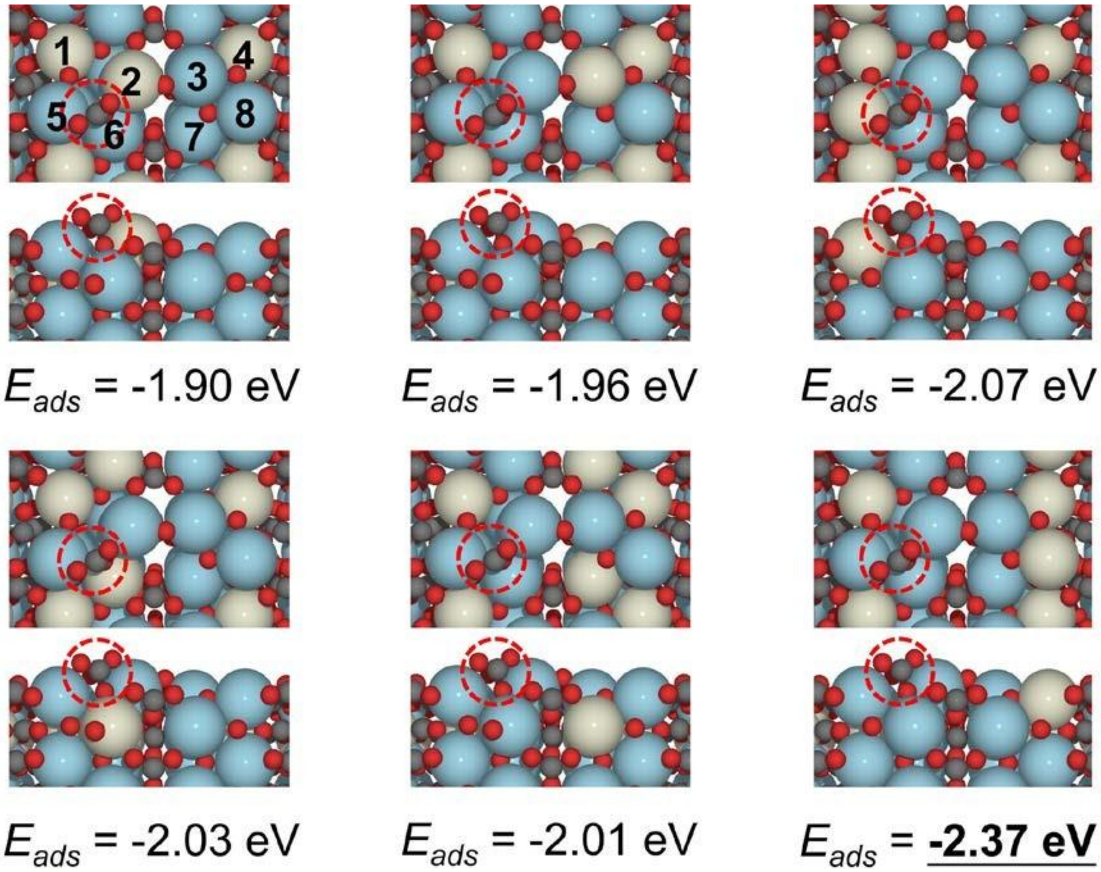


**Figure S8.** Possible models for 3Ce-doping and corresponding calculated $CO_2$ adsorption energies.

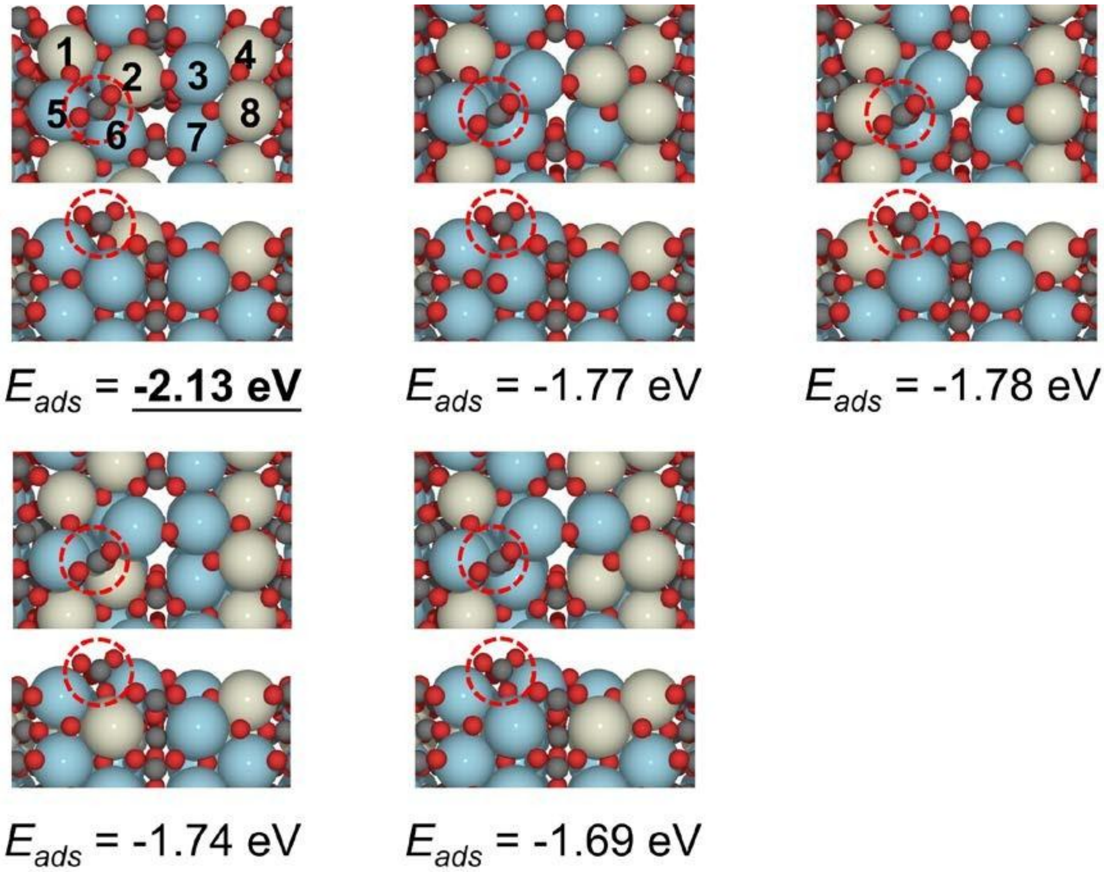


**Figure S9.** Possible models for 4Ce-doping and corresponding calculated $CO_2$ adsorption energies.

**Table S1.** Calculated $CO_2$ adsorption energies with randomly distributed Ce structures.

| 2Ce-doping | | 3Ce-doping | | 4Ce-doping | |
|---|---|---|---|---|---|
| Ce position[a] | $E_{ads}$ / eV | Ce position | $E_{ads}$ / eV | Ce position | $E_{ads}$ / eV |
| 3 and 8 | -1.29 | 4, 6 and 7 | -1.66 | 3, 4, 7, and 8 | -1.28 |
| 3 and 5 | -1.66 | 1, 4 and 7 | -2.05 | 2, 3, 5, and 8 | -1.78 |
| 5 and 8 | -1.60 | 1, 3 and 5 | -2.36 | 1, 3, 5, and 7 | -1.74 |
| 6 and 7 | -1.62 | | | 1, 2, 3, and 6 | -1.69 |
| 1 and 7 | -2.06 | | | 2, 4, 5, and 7 | -1.31 |
| 7 and 8 | -1.35 | | | 1, 2, 4, and 5 | -1.92 |
| 1 and 5 | -1.55 | | | 1, 2, 5, and 6 | -1.36 |
| 5 and 6 | -1.63 | | | 1, 3, 4, and 5 | -2.04 |
| 2 and 3 | -1.16 | | | | |

[a] See definition in Figure 11.
